# Inhibition of Vaginal Lactobacilli by a Bacteriocin-Like Inhibitor Produced by *Enterococcus faecium* 62-6: Potential Significance for Bacterial Vaginosis

**DOI:** 10.1080/10647440300025513

**Published:** 2003

**Authors:** Maureen C. Kelly, Michael J. Mequio, Vivien Pybus

**Affiliations:** Department of Biology Kalamazoo College 1200 Academy Street Kalamazoo MI 49006 USA

## Abstract

*Objective:* Bacterial vaginosis (BV) is characterized by a shift in vaginal tract ecology, which includes a decrease in the concentration and/or prevalence of facultative lactobacilli. Currently, mechanisms which could account for the disappearance of lactobacilli are not well understood. The objective of this study was to determine whether vaginal streptococci/enterococci can produce bacteriocin-like inhibitors antagonistic to vaginal lactobacilli.

*Methods:* Seventy strains of vaginal streptococci or enterococci were tested for antagonistic activities against vaginal lactobacilli using the deferred antagonism technique.

*Results:* One strain, *Enterococcus faecium* 62-6, which strongly inhibited growth of lactobacilli was selected for further characterization. The spectrum of inhibitory activity of strain 62-6 included Gram-positive organisms from the vaginal environment, although native lactobacilli from the same host were resistant to inhibitor action. Following growth inMRSbroth the strain 62-6 inhibitor was shown to be heat- (100℃, 30 minutes), cold- (4℃, less than 114 days) and pH- (4–7) stable. The sensitivity of inhibitor-containing supernatants to pepsin and α-chymotrypsin suggested an essential proteinaceous component. The inhibitor was sensitive to lipase but resistant to lysozyme. Dialysis of inhibitor-containing culture supernatants suggested a molecular mass greater than 12 000 Da. All physicochemical properties were consistent with its classification as a bacteriocin-like inhibitor. Kinetic assays demonstrated a sharp onset of inhibitor production coinciding with a concentration of 62-6 of 10^7^ cfu/ml, suggesting that production may be regulated by quorum sensing.

*Conclusions:* These results may have clinical significance as a novel mechanism to account for the decline of vaginal *Lactobacillus* populations and contribute to both the establishment and recurrence of BV.


*Infect Dis Obstet Gynecol 2003;11:147–156*

### Inhibition of vaginal lactobacilli by a bacteriocin-like inhibitor produced by *Enterococcus faecium* 62-6: potential significance for bacterial vaginosis

**Maureen C. Kelly, Michael J. Mequio and Vivien Pybus**

*Department of Biology, Kalamazoo College, 1200 Academy St., Kalamazoo, MI*

***Objective:*** Bacterial vaginosis (BV) is characterized by a shift in vaginal tract ecology, which includes a decrease in the concentration and/or prevalence of facultative lactobacilli. Currently, mechanisms which could account for the disappearance of lactobacilli are not well understood. The objective of this study was to determine whether vaginal streptococci/enterococci can produce bacteriocin-like inhibitors antagonistic to vaginal lactobacilli.

***Methods:*** Seventy strains of vaginal streptococci or enterococci were tested for antagonistic activities against vaginal lactobacilli using the deferred antagonism technique.

***Results:*** One strain, *Enterococcus faecium* 62-6, which strongly inhibited growth of lactobacilli was selected for further characterization. The spectrum of inhibitory activity of strain 62-6 included Gram-positive organisms from the vaginal environment, although native lactobacilli from the same host were resistant to inhibitor action. Following growth in MRS broth the strain 62-6 inhibitor was shown to be heat- (100°C, 30 minutes), cold- (4°C, less than 114 days) and pH- (4–7) stable. The sensitivity of inhibitor-containing supernatants to pepsin and $\alpha$-chymotrypsin suggested an essential proteinaceous component. The inhibitor was sensitive to lipase but resistant to lysozyme. Dialysis of inhibitor-containing culture supernatants suggested a molecular mass greater than 12 000 Da. All physicochemical properties were consistent with its classification as a bacteriocin-like inhibitor. Kinetic assays demonstrated a sharp onset of inhibitor production coinciding with a concentration of 62-6 of $10^7$ cfu/ml, suggesting that production may be regulated by quorum sensing.

***Conclusions:*** These results may have clinical significance as a novel mechanism to account for the decline of vaginal *Lactobacillus* populations and contribute to both the establishment and recurrence of BV.

Key words: BACTERIAL VAGINOSIS; BACTERIOCINS; *ENTEROCOCCUS*; VAGINAL LACTOBACILLI; MICROBIAL INTERACTIONS

Bacterial vaginosis (BV) is the most common vaginal tract infection seen in women of reproductive age in primary health care[1]. BV has a complex microbiology, characterized by an altered ecology of the vaginal tract which is reflected by the composition of the microflora. *Lactobacillus* populations, which are usually dominant in healthy women, are replaced by a polymicrobial group of organisms which includes the facultative organism *Gardnerella vaginalis*, Gram-positive and Gram-negative anaerobes, the genital mycoplasmas and sometimes *Mobiluncus* species[2]. During BV, total counts of aerobes and anaerobes are elevated 100- and 1000-fold, respectively, over normal levels[3]. However, the factor(s), either endogenous or exogenous, that initiate the shift in

Correspondence to: Vivien Pybus, PhD, Department of Biology, Kalamazoo College, 1200 Academy Street, Kalamazoo, MI 49006, USA. Email: vpybus@kzoo.edu

© 2003 The Parthenon Publishing Group 

the ecology of the vagina and result in the massive overgrowth of these microorganisms are not well-understood[2,4].

Interest in understanding the pathogenesis of BV stems from its clinical association with adverse pregnancy outcome[1,5], pelvic inflammatory disease[6], postoperative infections following gynecologic and obstetric surgeries[7] and increased risk of HIV acquisition via heterosexual intercourse[8–10]. While various antibiotic regimens for the treatment of BV are available, relapse rates are notoriously high[1,5]. Recurrence rates have been reported as high as 80% within a year of therapy[11] and 50% 5 to 9 years following treatment[12]. The failure of antibiotic treatment may reflect, in part, our limited understanding of the ecology of the vaginal tract including microbial interactions of significance during both health and BV.

One of the commonly-recognized, but poorly understood, features in the shift from a normal to a BV-associated flora is that the facultative lactobacilli lose their dominance as indicated by the decrease in their concentration and/or prevalence *in vivo*[1,2,4,7,13]. Results from one clinical study suggest that *Lactobacillus* populations are one of the first populations to decline in a sequence of population changes in the vagina leading to the establishment of BV[14]. Only a few studies have sought ecologically-based explanations, in particular microbial interactions, which could contribute to the decline in concentration and/or prevalence of facultative lactobacilli in the context of understanding the pathogenesis of BV. Notably, Tao and co-workers have reported the production of both phage (from vaginally- and environmentally-derived lactobacilli) and possible bacteriocins produced by lactobacilli from nonvaginal sources, which inhibit the growth of vaginal lactobacilli[15–17].

Sexual experience, especially exposure to a new sex partner or multiple sex partners, has been a strongly documented risk factor for BV[1]. Sexual contact provides an opportunity for microorganisms to be introduced into the vagina. Strains of streptococci and enterococci from many different sources have been well documented for their ability to produce bacteriocins or bacteriocin-like inhibitory substances[18–20]. Since these genera are components of the healthy vaginal microflora[21,22], they have the potential of establishing themselves if introduced into a new host. In addition, should they be bacteriocin producers, they have the potential of disrupting the microflora of their new host. With this in mind, the aim of the current study was to test streptococcal and enterococcal isolates of vaginal origin for the production of bacteriocin-like inhibitors antagonistic to the growth of vaginal lactobacilli. The discovery of inhibitor-producing strains with the potential of establishing themselves in a new host would be a novel mechanism to account for the decline of the vaginal lactobacilli, thus potentially paving the way for the establishment of a BV-associated microflora. This study reports the characterization of a bacteriocin-like inhibitor produced by a vaginally-derived isolate of *Enterococcus*, *E. faecium* strain 62-6, which was shown to be antagonistic to the growth of vaginal lactobacilli.

### MATERIALS AND METHODS

### Bacterial strains and culture conditions

Bacteria were isolated from discarded routine blood agar (BA) and chocolate agar culture plates obtained from both overtly healthy, including pregnant, and symptomatic women of menarchal age presenting for vaginal examination in physicians' offices in Kalamazoo County and sent to Bronson Hospital, Kalamazoo, MI. Vaginal cultures were plated on both BA and chocolate agar and cultivated in a humid atmosphere at 35°C in the presence of 5% (v/v) $CO_2$ (standard conditions of incubation) and provided to us by the Department of Clinical Microbiology, Bronson Hospital. Culture plates were accompanied by brief information including age of patient, whether or not she was pregnant, plus a brief description of the vaginal diagnosis and/or a summary of the main organisms present, as assessed by the Department of Clinical Microbiology. Patient names were kept confidential and not released to us.

Cultivation in liquid media was routinely carried out using 7 ml volumes of Lactobacilli MRS (MRS; Difco, Detroit, MI) broth or Brain Heart Infusion (BHI; Difco). BA containing 5%

(v/v) sheep blood (Presque Isle Cultures, Presque Isle, PA) and MRS agar (MRS broth containing 1.5% agar) were the solid cultivation media. Unless stated otherwise, routine cultivation was overnight (18–20 hours) under standard conditions of incubation. Anaerobic conditions were generated using the BBL Gas Pak Anaerobic System (Sparks, MD).

### Identification of bacteria

All isolates of vaginal bacteria were tentatively identified to genus level. Gram-positive, catalase-positive cocci were classified as belonging to the genus *Streptococcus* or *Enterococcus* and Gram-positive, catalase-positive elongated rods were considered *Lactobacillus* spp. *Lactobacillus* isolates 1-1, 4-1, 7-1, 9-1, 20-3, 33-4, 46-1 and 46-4 were identified as *L. acidophilus*, strain 62-5 as *L. casei* subsp. *rhamnosus* and strain 62-6 as *E. faecium* by the Michigan Department of Community Health, Lansing, MI. The enterococcal isolate 46-2 was identified as *E. faecalis* at Bronson Hospital. Stock cultures of each strain were prepared by the addition of glycerol (final concentration, 10% v/v) to overnight MRS broth cultures for the lactobacilli and BHI cultures for the streptococci and enterococci and stored at –80°C.

### The deferred antagonism technique for detection of bacteriocin-like inhibitors on solid media

This was essentially carried out according to Tagg and Bannister[23]. Seventy streptococcal and enterococcal strains of vaginal origin (potential producer strains) were grown on BA overnight as 1-cm-wide diametric streak cultures. Following the removal of visible growth, each plate was surface-sterilized by exposure to chloroform vapors, then air dried for at least 30 minutes. Thirty-two overnight cultures of *Lactobacillus* indicator bacteria, also of vaginal origin, were applied at right angles to the diametric streak cultures using a cotton-tipped swab. Plates were incubated then examined after 20 and then 44 hours' incubation for any zones of growth inhibition of the indicator bacteria.

### Detection of bacteriocin-like activity in liquid preparations

The well diffusion technique[24] was used to detect the inhibitory activity of liquid preparations, including culture supernatants, following addition of a standard volume of 100 μl to each well. Plates were surface-sterilized as above. Following application of indicator bacteria, plates were incubated for 20 and 44 hours before being examined for the presence of zones of inhibited growth in the vicinity of each well. The diameter of any zone of inhibition, including the 5 mm diameter of the well, was measured in mm. For both bacteriocin detection assays, indicator bacteria were cultured overnight in MRS broth.

### Stability of the anti-lactobacillus inhibitor produced by *E. faecium* 62-6

The heat, cold and enzyme stability of the inhibitor was tested using culture supernatants by conventional methods. For the enzyme susceptibility assays, culture supernatants were diluted 1:1 with the following enzymes prepared at a concentration of 2 mg/ml in phosphate (pH 7.0 and 8.0) or citrate buffer (pH 3.0) at the pH values indicated in parentheses: trypsin (Type II-S, porcine pancreas; 7.0), pepsin (porcine stomach mucosa; 3.0 and 7.0), proteinase K (*Tritrachium album*; 7.0), α-chymotrypsin (Type II, bovine pancreas; 8.0), catalase (bovine liver; 7.0), lysozyme (chicken egg white; 7.0) and lipase (*Candida rugosa*; 7.0). All enzymes were obtained from Sigma-Aldrich Company (St. Louis, MO). Exposure to catalase was also tested at the final concentrations of 30 μg/ml[25] and 540 μg/ml[26]. The pH sensitivity of the inhibitor-containing culture supernatants was tested using two methods. For the first method they were adjusted to pH values of 4.0, 5.0, 6.0 and 7.0 using a 1:1 dilution in either citrate (for pH 4.0 and 5.0) or phosphate (for pH 6.0 and 7.0) buffer, then assayed for inhibitory activity. For the second method, they were adjusted to pH values of either 4.0, 5.0, 6.0 and 7.0 using 0.1M HCl or NaOH, exposed for 1 hour at room temperature, then readjusted to the original pH before being assayed for inhibitory activity. Inhibitory activity was tested using the well diffusion assay.

### Molecular mass estimation of strain 62-6 inhibitor

Volumes, 3 ml, of inhibitor-containing supernatant were loaded into dialysis bags of molecular mass cut-off 3500 Da (Fisherbrand, Pittsburgh, PA), 8000 Da (Sigma-Aldrich) and 12 000 Da (Fisherbrand) and dialyzed against 300 ml volumes of citrate buffer, pH 5.0, at 4°C. The buffer solution was changed at least four times over 48 hours before assaying the dialyzed samples for inhibitory activity.

### Kinetics of strain 62-6 inhibitor production

The production of the bacteriocin-like inhibitor and concentration of the producer strain *E. faecium* 62-6 were monitored following inoculation of 20 ml volumes of MRS broth. Samples, 0.5 ml, were collected at 0, 1, 2, 3, 4, 5, 6, 10 and 24 hours following inoculation. Each sample was assayed by well diffusion for the presence of inhibitory activity and by plate count for the concentration of strain 62-6.

### Statistical analysis

Sigma Plot® (SPSS Science software products, Chicago, IL) was used for statistical analysis of the kinetic data. Throughout, all results represent the minimum of two independent experiments.

### RESULTS

### Screening for bacteriocin production against vaginal lactobacilli

Seventy vaginally-derived strains of bacteria belonging to the genera *Streptococcus* and *Enterococcus* were tested for their ability to produce bacteriocin-like inhibitors against 32 isolates of vaginal lactobacilli using the deferred antagonism technique on BA. Three strains (46-2, 62-6 and 78-1) inhibited the growth of at least half of the *Lactobacillus* indicator strains. Moreover, the zones of growth inhibition were reproducible and generally wide (extending up to 3 mm beyond the original producer streak). Of these three strong inhibitor producers, strain 62-6, identified as *E. faecium*, was selected for characterization of the nature of the inhibitory activity.

### Spectrum of inhibitory activity of *E. faecium* strain 62-6

Strain 62-6 was shown to inhibit the growth of 16 of the 32 vaginal *Lactobacillus* indicator strains tested (Table 1). The spectrum of inhibitory activity of strain 62-6 was then tested against a further 15 Gram-positive bacteria isolated from the vaginal environment as well as 15 Gram-positive and Gram-negative bacteria from various type culture collections (Table 1). Strain 62-6 was shown to inhibit the growth of four of the vaginally-derived bacteria, including three of five streptococci/enterococci and one of five

**Table 1** Spectrum of inhibitory activity of *E. faecium* strain 62-6 against bacteria isolated from the vaginal microflora and various bacteria from type culture collections, as detected using the deferred antagonism technique on BA

| *Indicator strain** | *No. of strains inhibited/ no. tested* |
|---|---|
| Vaginal microflora isolates: | |
| *Lactobacillus* spp. | 16/32 |
| *Streptococcus/Enterococcus* spp. | 3/5 |
| *Corynebacterium* spp. | 1/5 |
| *Staphylococcus* spp. | 0/5 |
| Type cultures: | |
| *Citrobacter freuendii* PI 239 | 0/1 |
| *Corynebacterium xerosis* ATCC 373 | 0/1 |
| *Edwardsiella tarda* PI 296 | 0/1 |
| *Enterobacter aerogenes* PI 341 | 0/1 |
| *Enterobacter cloacae* PI 343 | 0/1 |
| *Escherichia coli* ATCC 4157 | 0/1 |
| *Proteus mirabilis* ATCC 7002 | 0/1 |
| *Proteus vulgaris* ATCC 13315 | 0/1 |
| *Providencia alcalifaciens* PI 368 | 0/1 |
| *Pseudomonas aeruginosa* ATCC 10145 | 0/1 |
| *Serratia marsescens* PI 361 | 0/1 |
| *Staphylococcus aureus* ATCC 25923 | 0/1 |
| *Staphylococcus epidermidis* ATCC 14990 | 0/1 |
| *Streptococcus mutans* ATCC 25175 | 0/1 |
| *Streptococcus salivarius* | 0/1 |

*Sources: vaginal microflora and *Streptococcus salivarius* were isolated in this laboratory; PI, Presque Isle Cultures, Presque Isle, PA; ATCC, American Type Culture Collection, Manassas, VA

corynebacteria tested, but none of the vaginal staphylococci or any of the type cultures.

### Production and detection of the strain 62-6 inhibitor on blood-free culture media

The bacteriocin-like inhibitory activities of certain strains of streptococci and enterococci have been previously shown to be dependent on the presence of blood in the production and/or detection media[23,27]. The ability to produce inhibitors in blood-free, liquid cultivation media is advantageous for future physicochemical characterization assays of the inhibitor as well as its chemical purification. Therefore, the production of inhibitory activities by strain 62-6 was tested following growth in the blood-free liquid culture media MRS broth and BHI against a representative group of six out of the original 16 sensitive *Lactobacillus* indicator strains: 1-1, 4-1, 7-1, 9-1, 20-3, 33-4, plus the negative control strain 62-5 (Table 2). When MRS broth was used as the production medium and BA as the detection medium, MRS culture supernatants inhibited the growth of all sensitive *Lactobacillus* indicator strains, as we had observed in the original deferred antagonism test. In contrast, when BHI was used as the medium for production and BA for detection, inhibitory activities were limited to indicators 4-1 and 33-4. This was the same inhibitory pattern observed when MRS agar was used as the detection medium, irrespective of the production medium. Thus, strain 62-6 appears to produce certain metabolites during growth in MRS broth which could only be detected on BA by indicators 1-1, 7-1, 9-1 and 20-3 but not on MRS agar. The nature of these BA-dependent inhibitory effects was not further characterized as part of this study. From this point onwards *Lactobacillus* strain 4-1 was used as the representative positive control strain for detection of inhibitory activity by strain 62-6 on MRS agar as its sensitivity was independent of the presence of blood. Strain 62-5 was used as the negative control strain.

### Distinction from other antibacterial inhibitors

Control experiments were performed to exclude the possibility that the inhibitory activity produced by strain 62-6 was due to conditions of low pH alone, which can also cause antibacterial effects[28]. The pH of uninoculated MRS broth and BHI is 6.2 and 7.3, respectively, which reduces to pH values of 4.8 and 5.7 following growth of strain 62-6 under standard conditions of incubation. Uninoculated MRS broth and BHI were adjusted to pH values in the ranges 4.0–6.5 and 4.5–7.0, respectively. When assayed by well diffusion indicator strains 4-1 and 62-5 were resistant to all pH values tested, irrespective of growth medium.

As $H_2O_2$ production has been well documented to produce bacteriocin-like inhibitory effects[18], two different assays were carried out to exclude the possibility that the observed inhibition was due to $H_2O_2$ production. Firstly, as $H_2O_2$ is not produced anaerobically[18] strain 62-6 was grown in MRS broth under anaerobic conditions of incubation before testing for inhibitory activity. The pattern of inhibition was shown to be the same as that produced under standard (5% $CO_2$) incubation

**Table 2** Production of inhibitory activity in the blood-free liquid culture media, MRS broth and BHI, by *E. faecium* strain 62-6 against representative vaginal *Lactobacillus* indicator strains, as detected on BA and MRS agar by well diffusion

| *Agar detection medium* | *Liquid production medium* | *Growth inhibition* of* Lactobacillus *indicator strain* | | | | | | |
|---|---|---|---|---|---|---|---|---|
| | | *1-1* | *4-1* | *7-1* | *9-1* | *20-3* | *33-4* | *62-5* |
| BA | MRS | + | + | + | + | + | + | – |
| | BHI | – | + | – | – | – | + | – |
| MRS agar | MRS | – | + | – | – | – | + | – |
| | BHI | – | + | – | – | – | + | – |

*As assessed by measurement of the diameter (mm) of the zone of the inhibited growth of the indicator strain, inclusive of the well diameter (5 mm), where '+' indicates a diameter of the zone of growth inhibition of 7 mm or greater and '–' indicates a diameter of inhibited growth of less than 7 mm conditions (Table 3). To test this further, catalase was added to strain 62-6 culture supernatants prior to assay for inhibitory activity. Exposure to catalase had no demonstrable effect on inhibition production by strain 62-6 (Table 3), thus, the growth antagonistic activities by strain 62-6 appeared to be independent of $H_2O_2$ production.

### Assessment of the sensitivity of endogenous lactobacilli

In an attempt to assess the potential ecological significance of strain 62-6 inhibitor production *in vivo*, we tested whether a *Lactobacillus* isolate, strain 62-5, obtained from the same subject as the producer strain (62-6) was resistant or sensitive to the inhibitor. This was repeated using *Lactobacillus* strains 46-1 and 46-2 isolated from another woman who also carried an inhibitor-producing strain of *Enterococcus*, *E. faecalis* 46-2 (Table 4). For both women tested, the endogenous strains of lactobacilli were immune to the action of the producer strain from the same host when tested by both the deferred antagonism and well diffusion assays.

### Physicochemical characterization of strain 62-6 inhibitor

The physicochemical properties of the inhibitor produced by strain 62-6 were tested and the molecular mass estimated (Table 3). The inhibitor appeared heat stable following boiling for 30 minutes but not autoclaving, and was stable in MRS culture supernatants at 4°C for over 3 months. To test whether the inhibitor contained an essential proteinaceous component, inhibitor-containing supernatants were exposed to a panel of proteolytic agents. The inhibitor was sensitive to pepsin and to α-chymotrypsin but resistant to the proteases trypsin and proteinase K. In addition, it was resistant to lysozyme but sensitive to lipase. The strain 62-6 inhibitor was stable following exposure to conditions of acid pH, including pH values characteristic of the vagina during both health and BV (4.0 and 4.5, respectively). Inhibitory activity did not pass through a 0.22 μm cellulose membrane during filtration, which eliminated this procedure as a means of obtaining sterile, inhibitor-containing culture supernatants.

**Table 3** Physicochemical characterization of the inhibitor produced by *E. faecium* strain 62-6 against the sensitive *Lactobacillus* indicator strain 4-1 and the resistant strain 62-5. All assays were carried out following growth of strain 62-6 in MRS broth and assayed for inhibitory activity by well diffusion on MRS agar

| *Inhibitory activity of culture supernatants following* | *Growth inhibition* of indicator strain* | |
|---|---|---|
| | *4-1* | *62-5* |
| No treatment, positive control | + | – |
| Anaerobic incubation | + | – |
| Exposure to 50, 60, 80 or 100°C for 30 minutes | + | – |
| Storage at 4°C for < 114 days | + | – |
| Autoclaving, 121°C 15 minutes | – | – |
| Exposure to: | | |
| trypsin | + | – |
| pepsin | – | – |
| α-chymotrypsin | – | – |
| proteinase K | + | – |
| lysozyme | + | – |
| lipase | – | – |
| catalase | + | – |
| Exposure to pH 4–7 | + | – |
| Filtration through a 0.22 μm cellulose membrane | – | – |
| Dialysis in membranes of molecular mass cut-off: | | |
| 3500 | + | – |
| 8000 | + | – |
| 12 000 | + | – |

*As assessed by measurement of the diameter (mm) of the zone of the inhibited growth of the indicator strain, inclusive of the well diameter (5 mm), where '+' indicates a diameter of the zone of growth inhibition of 7 mm or greater and '–' indicates a diameter of inhibited growth of less than 7 mm

A preliminary estimate of the molecular mass following dialysis of culture supernatants in membranes with pore sizes up to 12 000 Da showed the retention of inhibitory activity in all three membranes, possibly indicating a mass greater than 12 000 Da.

### Kinetics of inhibitor production

The kinetics of inhibitor production by strain 62-6 are shown in Figure 1. Results from each of the three experiments consistently detected inhibitory activity at about 4 hours following inoculation, which coincided with mid to late log phase of growth. Interestingly, the onset of inhibitor production was sudden, corresponding to a concentration of strain 62-6 of ca. $10^7$ cfu/ml or greater. It also appeared rapid, as opposed to accumulating gradually in the culture supernatant over time. Such observations suggest that the regulation of inhibitor production may be subject to quorum sensing[29], where a bacterial population reaches a critical concentration before switching on (or off) production of a particular metabolic pathway.

**Table 4** Production of inhibitory activity by *E. faecium* strain 62-6 and *E. faecalis* strain 46-2 against strains of lactobacilli isolated from the same subject*, as assayed using both the deferred antagonism technique and the well diffusion assay, on BA

| | *Inhibition of endogenous* Lactobacillus *indicator strain* | | |
|---|---|---|---|
| *Producer strain* | *62-5* | *46-1* | *46-4* |
| 62-6 | – | NA | NA |
| 46-2 | NA | – | – |

*Strains 62-5 and 62-6 were isolated from a different woman than strains 46-1, 46-2 and 46-4. NA, not applicable

### DISCUSSION

While positive interactions between microbial populations of significance during BV have been demonstrated[30–32], the aim of this study was to examine the production of antagonistic substances by microbial populations of vaginal origin which could lead to the disappearance of the lactobacilli and potentially pave the way for the establishment of a BV-associated microflora. In 1961, Pohunek[33] briefly described enterococci isolated from women with a disturbed vaginal microflora that were antagonistic to the growth of vaginal lactobacilli, however the nature of the inhibitory activity was not investigated. James and Tagg[27] preliminarily reported one enterococci strain of vaginal origin, *E. faecalis* ssp. *liquefaciens* T142, which produced both blood-dependent and blood-independent inhibitors antagonistic to the growth of vaginal lactobacilli. Tao[16] described possible bacteriocins

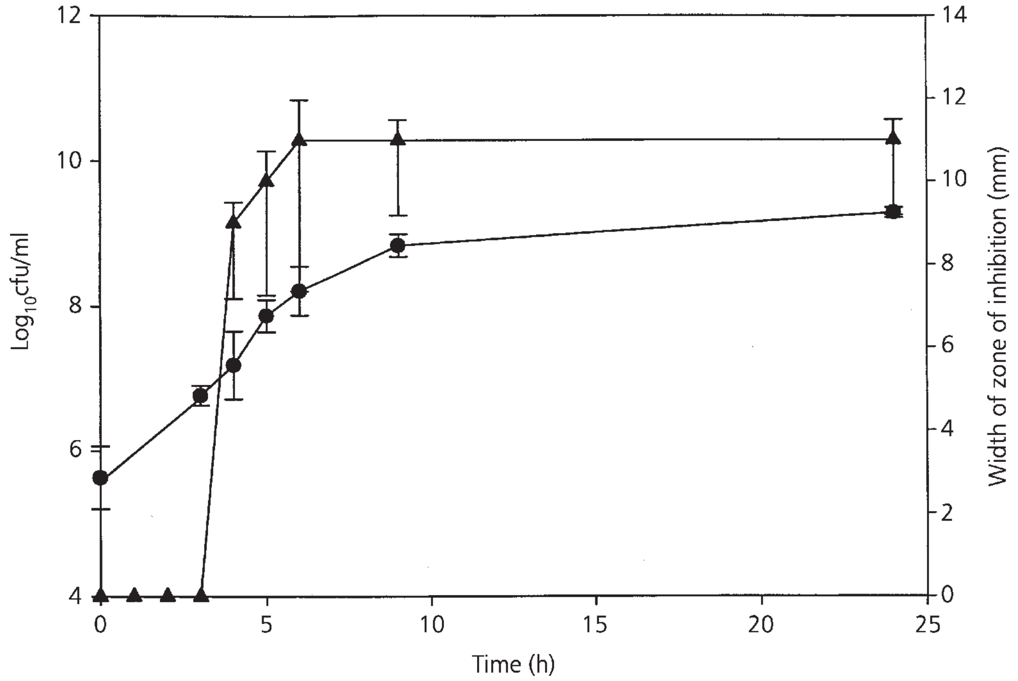

**Figure 1** Kinetics of inhibitor production over time. Results represent the means and standard deviations of three independent experiments. The concentration of *E. faecium* strain 62-6 (●) was determined by plate count on BA. The inhibitory activity against indicator strain 4-1 (▲) was determined using the well diffusion assay. The width of the zone of inhibition was obtained by subtracting the 5 mm diameter of the well from the diameter of the zone of inhibition produced by lactobacilli from nonvaginal sources which inhibited the growth of vaginal lactobacilli; however, these inhibitors have yet to be characterized in detail. With the exception of the aforementioned studies, the hypothesis that vaginally-derived streptococci (including enterococci) produce bacteriocins antagonistic to the growth of vaginal lactobacilli has not been addressed. In initial studies undertaken to address this hypothesis we tested 70 vaginal isolates of streptococci and enterococci for their ability to produce bacteriocin-like inhibitors against 32 vaginal lactobacilli using the deferred antagonism technique. This study reports the physicochemical characterization of the inhibitor produced by one strain, *E. faecium* 62-6, which strongly inhibited the growth of vaginal lactobacilli.

The spectrum of inhibitory activity of *E. faecium* strain 62-6 was essentially limited to Gram-positive organisms from the same ecological niche, including closely-related genera and vaginal lactobacilli. This finding is similar to that reported for the bacteriocin produced by *Streptococcus zymogenes* (now *E. faecalis* var. *zymogenes*)[34] and consistent with bacteriocins produced by other lactic acid bacteria, which often inhibit the growth of bacteria outside their own genus[19,20].

Characterization experiments were performed to determine the nature of the blood-independent inhibitory activity by strain 62-6 using inhibitor-containing MRS broth culture supernatants. We initially established that the inhibitory effect against sensitive indicator bacteria was not due to the production of acid conditions or hydrogen peroxide alone, effects that have often been reported to mimic bacteriocin-like inhibition[28]. We then demonstrated that the inhibitor was both heat, cold and pH stable, surviving exposure to temperatures in the range 50°C to 100°C for 30 minutes (but not autoclaving), storage at 4°C for at least 114 days and exposure to conditions in the pH range 4.0 to 7.0, respectively. The inhibitor adsorbed to cellulose membranes during filtration. These properties are consistent with those previously reported for bacteriocin-like inhibitors produced by lactic acid bacteria[18].

An essential defining characteristic of a bacteriocin is a proteinaceous component[18]. Inhibitor-containing MRS broth culture supernatants were sensitive following exposure to the proteolytic agents pepsin and α-chymotrypsin. These supernatants were, however, resistant to the proteinases trypsin and proteinase K. Differential sensitivity to a panel of proteases has been frequently reported for other bacteriocin-like inhibitors[18]. The inhibitor was resistant to lysozyme but sensitive to lipase, which may indicate that it contains an essential lipid component. The inability of the inhibitor to dialyze through membranes of molecular mass cut-off of 12 000 Da (or less) estimates that the molecular mass is greater than this. Future characterization assays using the chemically-purified inhibitor will allow us to more definitively characterize the physicochemical properties of this inhibitor. This will enable us to determine how this bacteriocin fits into the classification scheme as described by Klaenhammer[20], including whether it could be a class IV (complex) bacteriocin. However, all characteristics presented above for the inhibitor produced by strain 62-6 are consistent with those for previously-described bacteriocin-like inhibitors.

For two inhibitor-producing strains of *E. faecium*, strains 62-6 and 46-2, strains of lactobacilli isolated from the same subject were immune to inhibitor action. This observation suggests that the bacteriocin targets lactobacilli outside the original host while the endogenous strains are immune. Though preliminary, this finding is ecologically significant. It is also consistent as part of our original hypothesis that formed the basis for this study, that multiple sex partners, in particular the onset of a new sex partner, provides the opportunity for the acquisition of new strains of vaginal bacteria. If a bacteriocin-producing strain of *Enterococcus* from another host is introduced into the vaginal tract via sexual intercourse, it may be antagonistic to the growth of the endogenous lactobacilli present and cause their decline. This may, in turn, lead to the overgrowth of aerobes and anaerobes associated with BV.

One of the most striking findings during this study was the demonstration of inhibitor production following a minimum concentration of strain 62-6 of ca. $10^7$ cfu/ml, which suggests that regulation of inhibitor production is controlled via quorum sensing[29]. The presence of enterococci in the vaginal tract has been associated with a healthy vaginal microflora although levels do not always exceed $10^7$ cfu/ml[22]. However, if our *in vitro* observation applies *in vivo*, when present in high enough levels, bacteriocinogenic enterococci producing inhibitors antagonistic to the growth of vaginal lactobacilli may be one mechanism to account for the establishment of a BV-associated microflora.

This result may be of considerable clinical significance as one mechanism accounting for the high recurrence rate of BV following antibiotic therapy when the overgrowth of enterococci is a frequent, persistent problem. In clinical studies using topical clindamycin cream to treat BV, enterococci were reported to increase in prevalence from low (range, 0 to 11%) to high levels (range, 47 to 62%)[35,36] following therapy. Persistence of enterococci in approximately 50% of women 1 month after treatment for BV was reported by Hillier and co-workers[36]. While concentrations of persistent enterococci in the study reported by Hill and Livengood[35] did not exceed $10^7$ cfu/ml, the critical concentration at which we have shown inhibitor production to occur *in vitro*, it is conceivable that these levels could be reached in some subjects. Thus, for women who have been treated with antibiotics for BV and who become colonized with high concentrations of bacteriocinogenic strains of enterococci such as strain 62-6, bacteriocin production may prevent establishment of the normal microflora. This could be a novel mechanism contributing to the high recurrence rate of BV. Clinical studies addressing the prevalence, concentration and bacteriocinogenic potential of persistent enterococcal strains in recurrent BV subjects would address the significance of this as a mechanism to account for recurrent BV.

Understanding the interactions among microorganisms which comprise the normal vaginal microflora as well as factors which disrupt this ecological balance and lead to the establishment of BV has been proposed by several researchers[4,37,38]. Such information will solve some of the mysteries surrounding the pathogenesis of BV. Ultimately, the elucidation of such mechanisms will open new, ecologically-based procedures for the control of a syndrome where antibiotic treatment has so clearly been ineffectual[1].

### REFERENCES

1. Hillier S, Holmes KK. Bacterial vaginosis. In Holmes KK, Sparling PF, Mårdh PA, eds. *Sexually Transmitted Diseases*. New York: McGraw-Hill, 1999
2. Hill GB. The microbiology of bacterial vaginosis. *Am J Obstet Gynecol* 1993;169:450–4
3. Spiegel CA, Amsel R, Eschenbach D, *et al.* Anaerobic bacteria in nonspecific vaginitis. *N Engl J Med* 1980;303:601–7
4. Sobel JD. Bacterial vaginosis – an ecologic mystery. *Ann Intern Med* 1989;111:551–3
5. French JI, McGregor JA. Bacterial vaginosis. In Faro S, Soper DE, eds. *Infectious Diseases in Women*. Philadelphia, London, New York, St. Louis, Sydney, Toronto: WB Saunders, 2001.
6. Sweet RL. Role of bacterial vaginosis in pelvic inflammatory disease. *Clin Infect Dis* 1995;20 (Suppl 2):S271–5
7. Eschenbach DA. Bacterial vaginosis: emphasis on upper genital tract complications. *Obstet Gynecol Clin North Am* 1989;16:593–610
8. Sewankambo N, Gray RH, Wawer MJ, *et al.* HIV-1 infection associated with abnormal vaginal flora morphology and bacterial vaginosis. *Lancet* 1997;350:546–50
9. Cohen CR, Duerr A, Pruithithada N, *et al.* Bacterial vaginosis and HIV seroprevalence among female commercial sex workers in Chaing Mai, Thailand. *AIDS* 1995;9:1093–7
10. Warren D, Klein S, Sobel J, *et al.* A multicenter study of bacterial vaginosis in women with or at risk for human immunodeficiency virus infection. *Infect Dis Obstet Gynecol* 2001;9:133–41
11. Sobel JD. Long-term follow-up of patients with bacterial vaginosis treated with oral metronidazole and topical clindamycin. *J Infect Dis* 1993;167: 783–4
12. Boris J, Påhlson C, Larsson PG. Six years observation after successful treatment of bacterial vaginosis. *Infect Dis Obstet Gynecol* 1997;5:297–302
13. Amsel R, Totten PA, Spiegel CA, *et al.* Nonspecific vaginitis. Diagnostic criteria and microbial and epidemiologic associations. *Am J Med* 1983; 74:14–22
14. Rosenstein IJ, Morgan DJ, Sheehan M, *et al.* Bacterial vaginosis in pregnancy: distribution of bacterial species in different gram-stain categories of the vaginal flora. *J Med Microbiol* 1996;45:120–6
15. Pavlova SI, Kilic AO, Mou SM, Tao L. Phage infection in vaginal lactobacilli: an *in vitro* study. *Infect Dis Obstet Gynecol* 1997;5:36–44
16. Tao L, Pavlova SI, Mou SM, *et al*. Analysis of *Lactobacillus* products for phages and bacteriocins that inhibit vaginal lactobacilli. *Infect Dis Obstet Gynecol* 1997;5:244–51
17. Tao L, Pavlova SI. Vaginal *Lactobacillus* phage plaques and electron micrograph. *Infect Dis Obstet Gynecol* 1998;6:236
18. Tagg JR, Dajani AS, Wannamaker LW. Bacteriocins of Gram-positive bacteria. *Bacteriol Rev* 1976;40:722–56
19. Jack RW, Tagg JR, Ray B. Bacteriocins of Gram-positive bacteria. *Microbiol Rev* 1995;59: 171–200
20. Klaenhammer TR. Genetics of bacteriocins produced by lactic acid bacteria. *FEMS Microbiol Rev* 1993;12:39–86
21. Onderdonk AB, Wisseman KW. Normal vaginal microflora. In Elsner P, Martius J, eds. *Vulvovaginitis*. New York, Basel, Hong Kong: Marcel Dekker, 1993
22. Redondo-Lopez V, Cook RL, Sobel JD. Emerging role of lactobacilli in the control and maintenance of the vaginal bacterial microflora. *Rev Infect Dis* 1990;12:856–72
23. Tagg JR, Bannister LV. 'Fingerprinting' β-haemolytic streptococci by their production of and sensitivity to bacteriocine-like inhibitors. *J Med Microbiol* 1979;12:397–411
24. Hynes WL, Tagg JR. Production of broad-spectrum bacteriocin-like activity by group A streptococci of particular M-types. *Zentralbl Bakteriol Mikrobio Hyg [A]* 1985;259:155–64
25. McLean NW, McGroarty JA. Growth inhibition of metronidazole-susceptible and metronidazole-resistant strains of *Gardnerella vaginalis* by lacto-bacilli *in vitro*. *Appl Environ Microbiol* 1996;62: 1089–92
26. Tagg JR. Production of bacteriocin-like inhibitors by group A streptococci of nephritogenic M types. *J Clin Microbiol* 1984;19:884–7
27. James SM, Tagg JR. Streptococcal and entero-coccal inhibition of endocervical lactobacilli. *FEMS Microbiol Lett* 1987;41:321–6
28. Tagg JR, Read RSD, McGiven AR. Bacteriocin of a group A streptococcus: partial purification and properties. *Antimicrob Agents Chemother* 1973; 4:214–21
29. Winans SC, Bassler BL. Mob psychology. *J Bacteriol* 2002;184:873–83
30. Chen KCS, Forsyth PS, Buchanan TM, Holmes KK. Amine content of vaginal fluid from untreated and treated patients with nonspecific vaginitis. *J Clin Invest* 1979;63:828–35
31. Pybus V, Onderdonk AB. A commensal symbiosis between *Prevotella bivia* and *Peptostreptococcus anaerobius* involves amino acids: potential significance to the pathogenesis of bacterial vaginosis. *FEMS Immunol Med Microbiol* 1998; 22:317–27
32. Pybus V, Onderdonk AB. Evidence for a commensal, symbiotic relationship between *Gardnerella vaginalis* and *Prevotella bivia* involving ammonia: potential significance for bacterial vaginosis. *J Infect Dis* 1997;175:406–13
33. Pohunek M. Streptococci antagonizing the vaginal lactobacillus. *J Hyg Epidemiol Microbiol Immunol (Prague)* 1961;5:267–70
34. Brock TD, Davie JM. Probable identity of a group D hemolysin with a bacteriocine. *J Bacteriol* 1963;86:708–12
35. Hill GB, Livengood III CH. Bacterial vaginosis-associated microflora and effects of topical intravaginal clindamycin. *Am J Obstet Gynecol* 1994;171:1198–204
36. Hillier S, Krohn MA, Watts DH, *et al*. Micro-biologic efficacy of intravaginal cream for the treatment of bacterial vaginosis. *Obstet Gynecol* 1990;76:407–13
37. Pybus V, Onderdonk AB. Microbial interactions in the vaginal ecosystem, with emphasis on the pathogenesis of bacterial vaginosis. *Microbes Infect* 1999;1:285–92
38. Faro S. Bacterial vaginosis: the quest continues. *Infect Dis Obstet Gynecol* 2000;8:75

RECEIVED 02/20/03; ACCEPTED 07/30/03
